# The evolving landscape of biomarkers for systemic therapy in advanced hepatocellular carcinoma

**DOI:** 10.1186/s40364-025-00774-2

**Published:** 2025-04-12

**Authors:** Xinyu Guo, Zhongwei Zhao, Lingyi Zhu, Shuang Liu, Lingling Zhou, Fazong Wu, Shiji Fang, Minjiang Chen, Liyun Zheng, Jiansong Ji

**Affiliations:** 1 Zhejiang Engineering Research Center of Interventional Medicine Engineering and Biotechnology, School of Medicine, Lishui Hospital, Zhejiaing University, Lishui, 323000 China; 2 Department of Radiology, The Fifth Affiliated Hospital of Wenzhou Medical University, Lishui Central Hospital, Lishui, 323000 China; 3 The 2nd Clinical Medical College, Zhejiang Chinese Medical University, Hangzhou, 310000 China; 4 Zhejiang Key Laboratory of Imaging and Interventional Medicine, The Fifth Affiliated Hospital of Wenzhou Medical University, Lishui, Zhejiang 323000 China

**Keywords:** Advanced hepatocellular carcinoma, Systemic therapy, Molecular targeted therapy, Immune checkpoint inhibitor, Biomarker

## Abstract

Hepatocellular carcinoma (HCC) remains one of the most prevalent and deadliest cancers. With the approval of multiple first- and second-line agents, especially the combination therapies based on immune checkpoint inhibitor (ICI) regimens, the landscape of systemic therapy for advanced HCC (aHCC) is more diverse than ever before. The efficacy of current systemic therapies shows great heterogeneity in patients with aHCC, thereby identifying biomarkers for response prediction and patient stratification has become an urgent need. The main biomarkers for systemic therapy in hepatocellular carcinoma are derived from peripheral blood, tissues, and imaging. Currently, the understanding of the clinical response to systemic therapy indicates unequivocally that a single biomarker cannot be used to identify patients who are likely to benefit from these treatments. In this review, we provide an integrated landscape of the recent development in molecular targeted therapies and ICIs-based therapies, especially focusing on the role of clinically applicable predictive biomarkers. Additionally, we further highlight the latest advancements in biomarker-driven therapies, including targeted treatments, adoptive cell therapies, and bispecific antibodies.

## Introduction

**Fig. 1:**
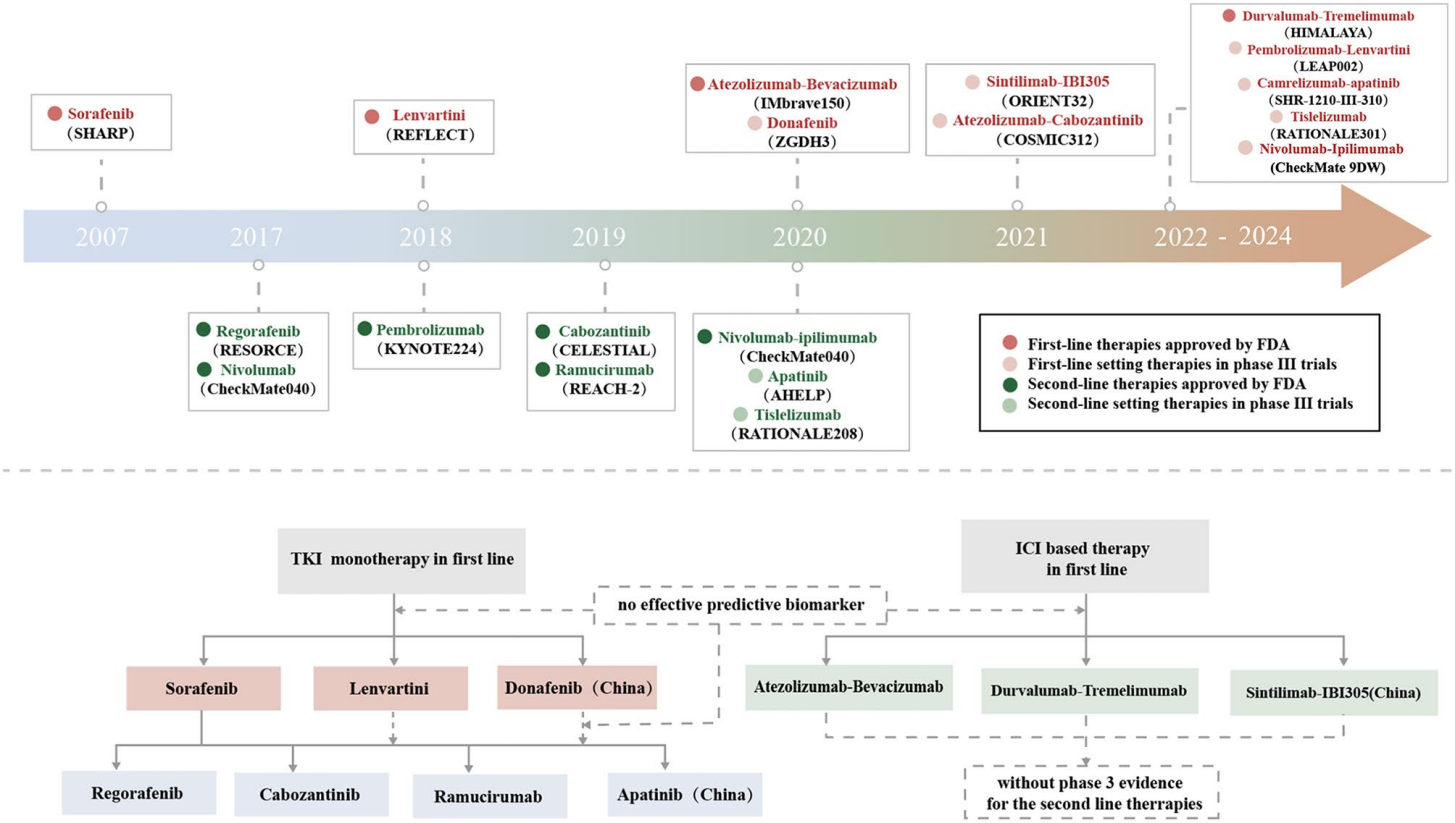
The landscape and sequential strategy of systemic therapy for patients with aHCC

Hepatocellular carcinoma (HCC) is one of the most common malignant tumors and the third leading cause of cancer-related deaths worldwide [1]. However, the 5-year survival rate of HCC is only 5–30% worldwide [1]. The management of cancer has been dramatically changed by systemic treatments using molecular targeted and immune therapies [2]. With the approval of multiple first- and second-line agents (Tables 1 and 2), especially the establishment of immune checkpoint inhibitors (ICIs)-based therapies, the landscape of systemic therapy for advanced HCC (aHCC) has become more diverse than ever (Fig. 1) [2]. The efficacy of current systemic therapies exhibits significant heterogeneity in patients with aHCC, highlighting the urgent need to identify biomarkers for response prediction and patient stratification of prognosis [3]. Nevertheless, biomarker research in HCC remains in its early stages. While no single biomarker is fully validated, alpha-fetoprotein (AFP) and ctDNA have shown promise in stratifying patients [3]. Studies aimed at identifying possible predictive biomarkers for systemic treatments including combination therapies in HCC have recently commenced. Notably, numerous novel therapeutic approaches based on biomarkers with the potential to offer improved treatment effects in patients with aHCC have also been proposed and developed [3, 4].

**Table 1:** The molecular targeted therapies approved for aHCC

Trial name	Setting	Agent	Target	Results	Approved	Summary
SHARP (Global)	1 L (*N* = 602)	Sorafenib vs. Placebo	VEGFR1–3, PDGFR, RAF, KIT	OS: 10.7 vs. 7.9 months (HR 0.69, *P* < 0.001)	FDA (2007)	The first targeted drug approved for aHCC was a multikinase inhibitor, which ushered in the era of targeted therapy for HCC
REFLECT (Global)	1 L (*N* = 954)	Lenvatinib vs. Sorafenib	VEGFR1–3, PDGFR, FGFR1–4, RET	ORR: 18.8% vs. 6.5% PFS: 7.3 vs. 3.6 months (HR 0.65, *P* < 0.0001) OS: 13.6 vs. 12.3 months (HR 0.92, non-inferiority)	FDA (2018)	Lenvatinib is not inferior to sorafenib in efficacy, especially in Asian populations and patients with HBV-related HCC
ZGDH3 (China)	1 L (*N* = 668)	Donafenib vs. Sorafenib	VEGFR1–3, PDGFR, RAF	ORR: 4.6% vs. 2.7% PFS: 3.7 vs. 3.6 months (HR 0.91, *P* = 0.057) OS: 12.1 vs. 10.3 months (HR 0.83, *P* = 0.0245)	NMPA (2020)	A small-molecule drug, which is a modified variant of sorafenib, exhibits comparable efficacy while demonstrating superior safety
RESORCE (Global)	2 L (*N* = 567)	Regorafenib vs. Placebo	VEGFR1–3, PDGFR, FGFR1–2, RAF	ORR: 29.8% vs. 11.3% PFS: 6.9 vs. 4.3 months (HR 0.65, *P* < 0.001) OS: 19.2 vs. 13.4 months (HR 0.66, *P* < 0.001)	FDA (2017)	As a second-line treatment, it is suitable for patients who have failed sorafenib treatment and significantly prolongs survival
CELESTIAL (Global)	2 L (*N* = 707)	Cabozantinib vs. Placebo	VEGFR1–3, MET, RET	ORR: 3.8% vs. 0.4% PFS: 5.2 vs. 1.9 months (HR 0.44, *P* < 0.001) OS: 10.2 vs. 8.0 months (HR 0.76, *P* = 0.005)	FDA (2019)	Multi-target inhibitors, which have dual effects on angiogenesis and tumor microenvironment, are suitable for treated patients
REACH-2 (Global)	2 L (*N* = 292)	Ramucirumab vs. Placebo	VEGFR2	ORR: 4.6% vs. 1.1% PFS: 2.8 vs. 1.6 months (HR: 0.45, *P* < 0.0001) OS: 8.5 vs. 7.3 months (HR 0.71, *P* = 0.0199)	FDA (2019)	The second-line therapeutic agents for patients with elevated AFP levels can specifically target the VEGFR2 pathway
AHELP (China)	2 L (*N* = 400)	Apatinib vs. Placebo	VEGFR2, KIT, RET, SRC	ORR: 4.6% vs. 1.1% PFS: 2.8 vs. 1.6 months (HR: 0.45, *P* < 0.0001) OS: 8.5 vs. 7.3 months (HR 0.71, *P* = 0.0199)	NMPA (2020)	Chinese-originated small molecule antiangiogenic drug, appropriate for second-line therapy

aHCC, advanced HCC; L, line; VEGFR, vascular endothelial growth factor receptor; PDGFR, platelet-derived growth factor receptor; KIT, kinase insert domain receptor; MET, mesenchymal-epithelial transition factor; RET, RET proto-oncogene; PD-1, programmable death-; ORR, objective response rate; HR, hazard ratio; PFS, progression free survival; OS, overall survival; FDA, Food and Drug Administration; NMPA, National Medical Products Administration

**Table 2:** The immune checkpoint inhibitors approved for aHCC

Trial name	Setting	Agent	Target	Results	Approved	Summary
IMbrave150 (Global)	1 L (*N* = 501)	Atezolizumab + bevacizumab vs. Sorafenib	PD-L1 + VEGFA	ORR: 29.8% vs. 11.3% PFS: 6.9 vs. 4.3 months (HR 0.65, *P* < 0.001) OS: 19.2 vs. 13.4 months (HR 0.66, *P* < 0.001)	FDA (2020)	The synergistic combination of immunotherapy and anti-angiogenesis therapy demonstrates markedly superior efficacy compared to sorafenib in first-line treatment
ORIENT32 (China)	1 L (*N* = 571)	Sintilimab + IBI305 vs. Sorafenib	PD-1 + VEGFA	ORR: 29.8% vs. 11.3% PFS: 6.9 vs. 4.3 months (HR 0.65, *P* < 0.001) OS: 19.2 vs. 13.4 months (HR 0.66, *P* < 0.001)	NMPA (2021)	The combination of PD-1 inhibitors and VEGF inhibitors has been particularly prominent in improving ORR and prolonging PFS, especially in patients with HBV-related HCC
HIMALAYA (Global)	1 L (*N* = 782)	Durvalumab + Tremelimumab vs. Sorafenib	PD-L1 + CTLA4	ORR: 20.1% vs. 5.1% PFS: 3.8 vs. 4.1 months (HR 0.90) OS: 16.4 vs. 13.8 months (HR 0.78, *P* = 0.0035)	FDA (2022)	Dual immune checkpoint combination therapy enhances the anti-tumor immune response through simultaneous blockade of CTLA-4 and PD-L1
CARES-310 (Global)	1 L (*N* = 543)	Camrelizumab + apatinib vs. Sorafenib	PD-1 + VEGFR2, KIT, RET, SRC	ORR:25.4% vs. 5.9% PFS:5.6 vs. 3.7months (HR 0.52, *P* < 0.001)) OS:22.1 vs. 15.2 months (HR 0.62, *P* < 0.001))	NMPA (2022)	The first successful phase III study of a PD-1 inhibitor combined with small molecule antiangiogenic drugs improves ORR and PFS in aHCC patients, with tolerable safety
KEYNOTE-394 (Asia)	2 L (*N* = 453)	Pembrolizumab vs. Placebo	PD1	ORR: 13.7% vs. 1.3% PFS: 2.6 vs. 2.3 months (HR 0.74, *P* = 0.0032) OS: 14.6 vs. 13.0 months (HR 0.79, *P* = 0.0180)	FDA (2018)	PD-1 inhibitors, which have demonstrated a high ORR, are suitable for treated patients
CheckMate040 armA (Global)	2 L (*N* = 50)	Nivolumab + Ipilimumab	PD1 + CTLA4	ORR:32% OS:22.8 months	FDA (2020)	The first FDA-approved dual immunotherapy for aHCC, particularly for patients who have failed prior treatments or cannot tolerate other options
NCT02989922	2 L (*N* = 217)	Camrelizumab	PD1	ORR:14.7% PFS:2.1 months OS:13.8 months	NMPA (2020)	As a self-developed Chinese PD-1 inhibitor, it significantly prolongs OS in second-line treatment with unique side effect of reactive cutaneous capillary hyperplasia for predicting efficacy
RATIONALE 208	2 L (*N* = 235)	Tislelizumab	PD1	ORR:13.3% PFS:2.7 months OS:13.2 months	NMPA (2020)	A structurally optimized PD-1 inhibitor demonstrates high efficacy, durability, and good safety in treated aHCC, particularly in Asian patients

aHCC, advanced HCC; PD-1, programmable death-; CTLA4, cytotoxic T-lymphocyte associated protein 4; ORR, objective response rate; HR, hazard ratio; PFS, progression free survival; OS, overall survival; FDA, Food and Drug Administration; NMPA, National Medical Products Administration

**Fig. 2:**
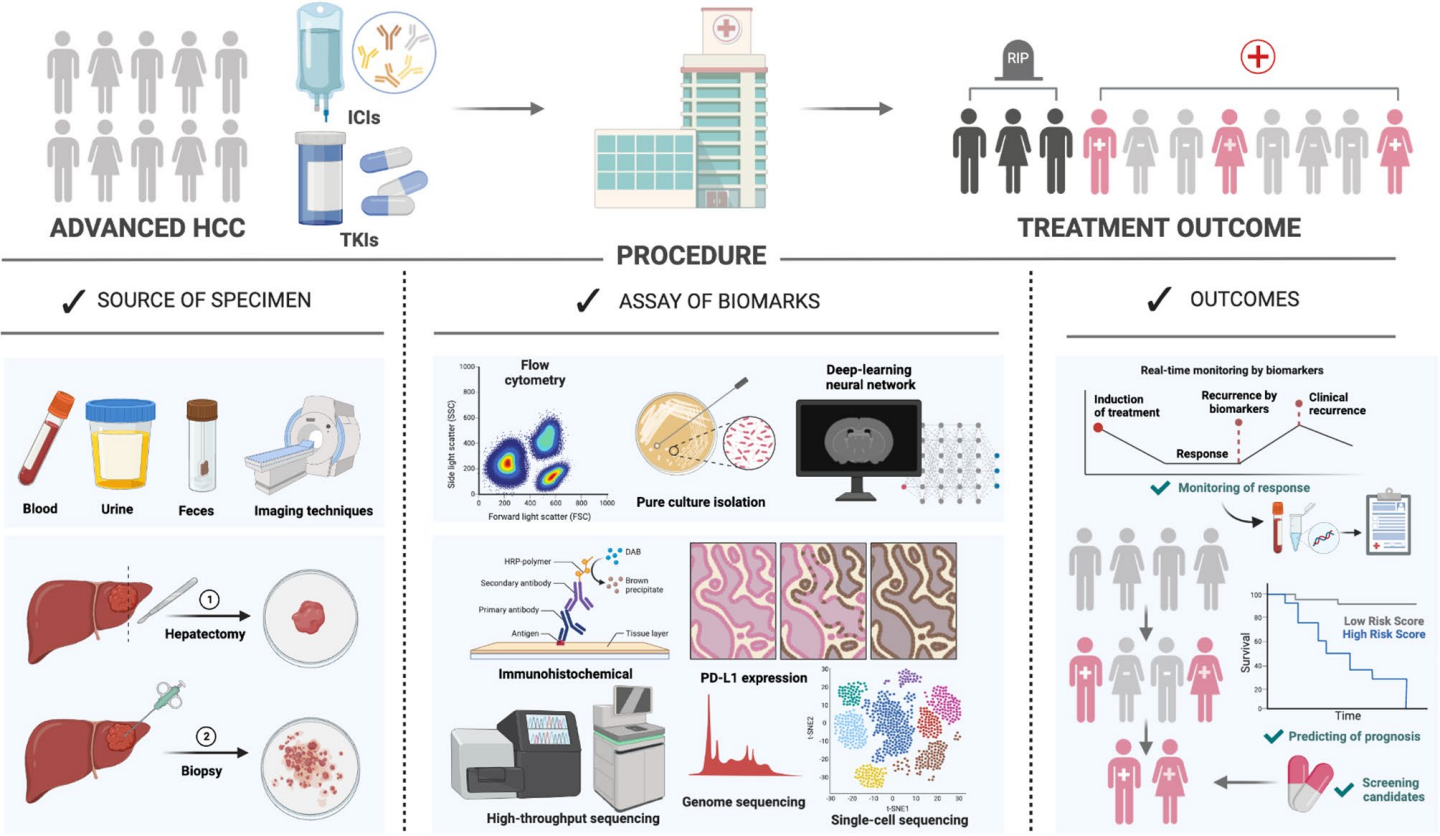
Procedure of biomarker exploration for systemic therapy of HCC. Biomarker exploration procedure for systemic therapy of HCC include collection of samples, assay of biomarkers and analysis of outcomes. Specimens sources included noninvasive and invasive methods. Researchers have analyzed specimen through a variety of techniques to predict the efficacy and prognosis of systemic therapy for HCC

**Fig. 3:**
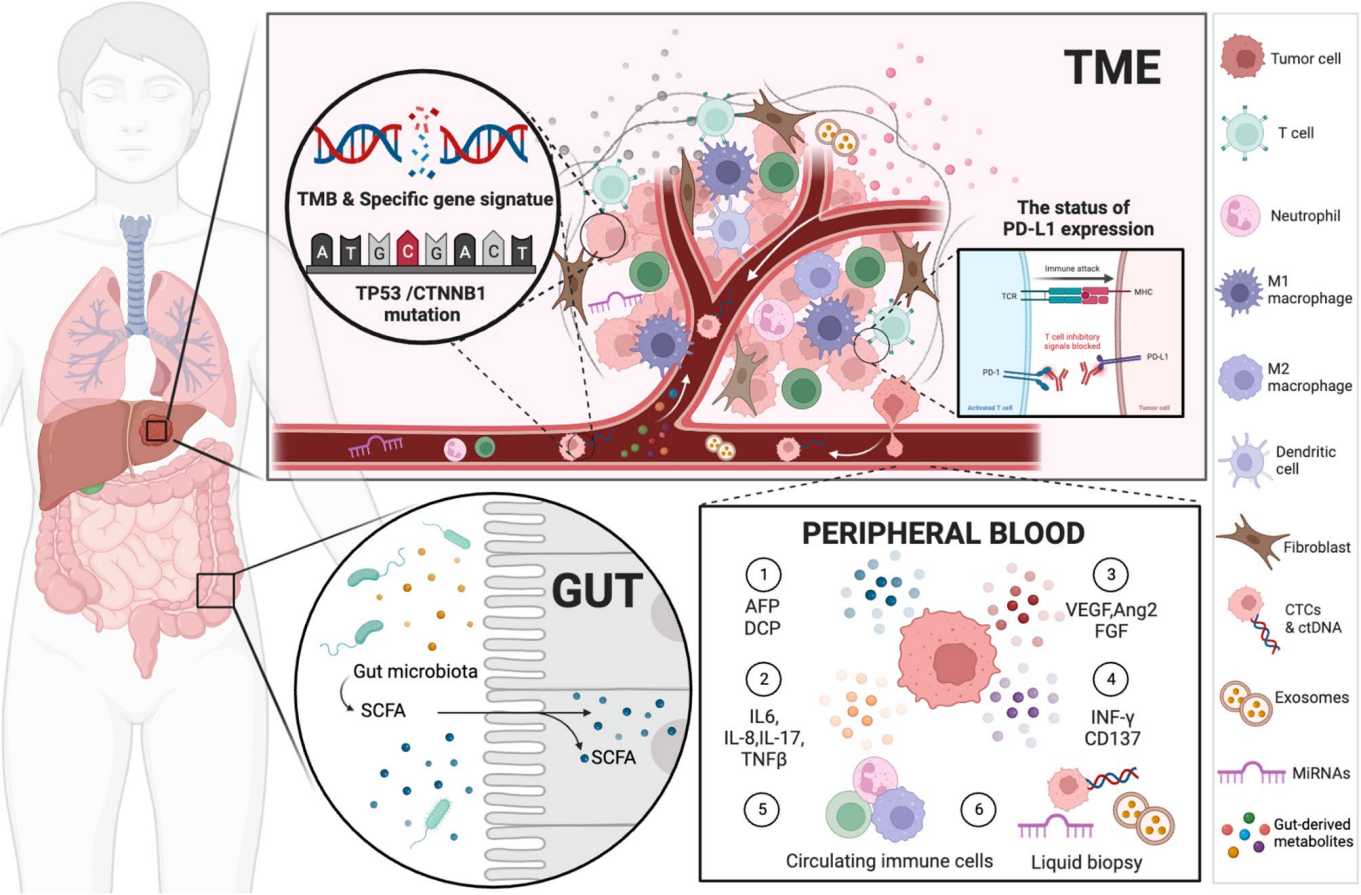
Overview of predictive or prognostic biomarkers for systemic therapies in HCC. Predictive or prognostic biomarkers for systemic therapies in HCC include peripheral blood biomarkers, liquid-biopsy, tumor tissue-associated biomarkers, and gut microbiota. AFP, alpha-fetoprotein; DCP, des-γ-carboxy prothrombin; VEGF, vascular endothelial growth factor; Ang, angiopoietin; FGF, fibroblast growth factor; SCFA, short-chain fatty acids; INFγ, interferon-γ; IL, interleukin; TNFβ, transforming growth factor-β; miRNA, micro RNA; CTC, circulating tumor cells; ct DNA, circulating tumor DNA; PD-L1, programmed cell death-ligand 1; TMB, tumor mutational burden; TME, tumor microenvironment

In this review, we provide an integrated landscape of the recent development in targeted therapies and ICIs based therapies, especially focusing on the role of clinically applicable predictive biomarkers from multiple dimensions (Tables 3 and 4; Figs. 2 and 3). Additionally, we further highlight the latest advancements in biomarker-driven therapies, including targeted treatments, adoptive cell therapies, and bispecific antibodies (Fig. 3).

**Table 3:** Predictive biomarkers for HCC targeted therapies in pivotal clinical trials

Trial name	Treatment	BEP	Specimen	Assay	Biomarker	Outcome
SHARP	Sorafenib (1 L)	*N* = 491 (baseline) *N* = 305 (post-treatment)	Peripheral blood	ELISA	Plasma Ang2,VEGF	Plasma Ang2 and VEGF were independent predictors of survival in patients with aHCC. None of 10 selected biomarkers was significantly associated with response to sorafenib.
REFLECT	Lenvatinib (1 L)	*N* = 477	Peripheral blood	ELISA	VEGF, ANG2, FGF19, FGF21, and FGF23	Higher baseline levels of VEGF, FGF21, and ANG2 may be prognostic for shorter OS. Higher baseline FGF21 may be predictive for longer OS with lenvatinib compared with sorafenib.
		*N* = 58	Tumor tissue	RNA seq	Angiogenesis and growth factor signaling pathway genes	Angiogenesis and growth factor signaling pathway genes identified three subgroups in HCC. One subgroup (high FGF ligand) showed longer OS compared with the other subgroups.
RESORCE	Regorafenib (2 L)	*N* = 499	Peripheral blood	Multiplex immunoassay	Plasma proteins	5 decreased baseline plasma protein (Ang 1, cystatin B, LAP TGFβ1, LOX-1, and MIP-1) were significantly associated with increased overall survival time after regorafenib treatment.
		*N* = 349		PCR	Plasma miRNAs	9 plasma miRNAs (MIR30A, MIR122, MIR125B, MIR200A, MIR374B, MIR15B, MIR107, MIR320, and MIR645) was significantly associated with overall survival time with regorafenib.
		*N* = 17	Tumor tissue	NGS	CTNNB1 mutations and VEGFA amplification	49 somatic aberrations in 27 oncogenes and tumor suppressor genes were revealed. CTNNB1 mutations were detected in 3 of 10 progressors and VEGFA amplification in 1 of 7 responders.
		*N* = 46		RNA seq	Inflammation gene expression	Low and medium/high immune cell groups identified by immune cell scores were not associated with the mOS or median TTP
CELESTIAL	Cabozantinib (2 L)	*N* = 707	Peripheral blood	ELISA	Serum AFP	AFP response was independently associated with longer OS. The optimal cutoff for association with OS in the cabozantinib arm was ≤ 0% change in AFP at week eight.
REACH-2	Ramucirumab (2 L)	*N* = 292	Peripheral blood	ELISA	Serum AFP	Patients with AFP concentrations of at least 400 ng/mL who had previously received sorafenib showed improved OS.

L, line; BEP, biomarker evaluable population; AFP,αfetoprotein; RNA seq, transcriptome sequencing; NGS, next generation sequencing; Ang2, Angiopoietin-2; VEGF, vascular endothelial growth factor; FGF, fibroblast growth factor; OS, overall survival; miRNAs, micro RNA

**Table 4:** Predictive biomarkers for HCC immunotherapy in pivotal clinical trials

Trial name	Treatment	BEP	Specimen	Assay	Biomarker	Results
KEYNOTE-224	Pembrolizumab (2 L)	*N* = 52	Tumor tissue	IHC	Baseline PD-L1 expression	PD-L1 defined by CPS was associated with response, PD-L1 defined by TPS was not associated with response.
CheckMate 459	Nivolumab (2 L)	*N* = 743	Tumor tissue	IHC	Baseline PD-L1 expression	Patients with PD-L1 ≥ 1% treated with nivolumab was found to be associated with longer median OS in those treated with sorafenib.
CheckMate 040	Nivolumab + ipilimumab (2 L)	*N* = 145	Tumor tissue	ICH	Baseline PD-L1 expression	Baseline PD-L1 expression was not associated with treatment response.
CheckMate 040	Nivolumab (1–2 L)	*N* = 44 (Escalation cohort) *N* = 174 (Expansion cohort)	Tumor tissue	IHC	Baseline PD-L1 expression	Baseline PD-L1 expression was not associated with treatment response.
		*N* = 195(PD-L1) *N* = 178(PD-1)	Tumor tissue	IHC	Baseline PD-L1 and PD-1 expression	Both were associated with improved OS. PD-1, but not PD-L1, was associated with ORR.
		*N* = 189(CD3) *N* = 192(CD8)		IHC	Baseline CD3 + and CD8 + TILs	CD3 + or CD8 + TILs exhibited a trend towards improved OS.
		*N* = 189(CD4) *N* = 190(FOXP3)		IHC	Baseline CD4 + and FOXP3 + TILs	CD4 + and FOXP3 + TILs were not associated with OS.
		*N* = 135		IHC	Baseline CD68 + and CD63 + TILs	CD68 + and CD63 + TILs were not associated with OS.
		*N* = 37		RNA seq	inflammation signatures	Inflammatory signature consisting of CD274 (PD-L1), CD8A, LAG3, STAT1 was associated with both improved objective response rate and overall survival.
		*N* = 242(NLR) *N* = 243(PLR)	Peripheral blood	Flow cytometry	Baseline NLR and PLR	Lower NLR and PLR were found to be associated with PR and CR on nivolumab.
		*N* = 149		ELISA	Baseline serum AFP	Low AFP (< 400 µg/L) showed a numerical (although not statistically significant) association with response.
GO 30,140 and IMbrave 150	Atezolizumab +Bevacizumab (1 L)	GO 30,140 (*N* = 59) IMbrave 150 (*N* = 150)	Peripheral blood	ELISA	Serum AFP	≥ 75% decrease or ≤ 10% increase in AFP levels at 6 weeks after combined therapy was significantly associated with improved PFS and OS.
		GO30140(*N* = 90); IMbrave150(Atezo plus Beva, *N* = 119; Sora, *N* = 58)	Tumor tissue	IHC	Baseline PD-L1 expression	The expression of PD-L1 was higher in responders; High expression of PD-L1 showed improved PFS and OS when treated with atezolizumab + bevacizumab compared to sorafenib.
		GO30140 (*N* = 90)	Tumor tissue	IHC	Immune subsets	High presence of immune subsets (CD8 + and CD4 + T cells, Tregs, B cells, and DCs) was associated with better response and longer PFS.
		IMbrave150 (Atezo plus Beva, *N* = 119; Sora, *N* = 58)	Tumor tissue	IHC	Intratumoral CD8 + T cells	Patients with a high density of intratumoral CD8 + T cells exhibited prolonged OS and PFS with atezolizumab plus bevacizumab, as compared to sorafenib.
		GO30140 (*N* = 76); IMbrave150 (Atezo plus Beva, *N* = 119; Sora, *N* = 58)	Tumor tissue	WES	TMB	Patients with high TMB were associated with higher ORR than those with low or median TMB in the GO30140 arm A, but TMB was not associated with PFS.
		IMbrave150 (Atezo plus Beva, *N* = 85; Sora, *N* = 45)	Tumor tissue	WES	TERT promoter mutation	Patients with TERT promoter mutation showed longer PFS and OS in the combination therapy group than in the sorafenib group.
					CTNNB1 mutation	Patients with wild-type CTNNB1 showed greater treatment effects and longer PFS from atezolizumab + bevacizumab vs. sorafenib than those with CTNNB1 mutations.
		GO30140 (*N* = 90); IMbrave150 (Atezo plus Beva, *N* = 119; Sora, *N* = 58)	Tumor tissue	RNA seq	ARBS and Teff	Higher expression of ABRS and Teff had better treatment response and longer PFS; High expression of ABRS or the Teff signature showed improved PFS and OS when treated with atezolizumab + bevacizumab vs. sorafenib.
					Treg/Teff	Low ratio of Treg/Teff signatures was associated with improved PFS and OS with atezolizumab + bevacizumab vs. sorafenib.

L, line; BEP, biomarker evaluable population; IHC, immunohistochemistry; OS, overall survival; PFS, progression free survival; WES, whole-exome sequencing; NLR, neutrophil-lymphocyte ratio; PLR, platelet-lymphocyte ratio; AFP, αfetoprotein; PD-L1, Programmed cell death 1 ligand 1; TMB, tumor mutational burden; Treg, regulatory T cell; Teff, T effector cell; TERT, telomerase reverse transcriptase

### Circulating biomarkers in peripheral blood

#### AFP as a Circulating biomarker

AFP is the most widely used and accepted serum tumor biomarker in HCC. The overexpression of AFP reflects more tumor aggressiveness and burden of HCC. Baseline AFP ≥ 400 ng/mL is a prognostic biomarker as well as a stratification biomarker for the most phase III clinical studies in aHCC [5–8]. Several studies have shown that changes in AFP levels are closely related to the response of patients with aHCC to systemic therapy, such as targeted therapy and immunotherapy. In the context of targeted drug therapies (including sorafenib, lenvatinib, cabozantinib, and regorafenib), patients exhibiting a reduction in AFP levels of ≥ 20% typically demonstrate prolonged progression-free survival (PFS) and overall survival (OS) [5, 9]. In the treatment of PD-1/PD-L1 inhibitors, such as nivolumab, pembrolizumab, patients with significantly reduced AFP levels tend to show a higher objective response rate (ORR) and longer survival [10]. The dynamic change of AFP was used as an early predictor of efficacy. The dynamic changes in AFP levels during the early treatment phase (e.g., 4–8 weeks), such as a decrease of ≥ 50%, can serve as a sensitive indicator for the early prediction of treatment efficacy and assist clinicians in promptly adjusting the treatment strategy [11]. The baseline AFP level is an independent predictor of the prognosis of patients with advanced hepatocellular carcinoma. High AFP level (≥ 400 ng/mL) is associated with more aggressive tumor, higher risk of vascular invasion, higher rate of distant metastasis, and poor prognosis. In the REFLECT trial, median OS was significantly prolonged in patients with lower baseline AFP levels [12]. Similarly, patients with ≥ 75% reduction in AFP levels had significantly better median OS than those with < 75% reduction in the IMbrave150 trial [8]. AFP levels can be used to screen patients suitable for specific treatment regimens. For example, patients with AFP ≥ 400 ng/mL had a more significant survival benefit with Ramucirumab [6].

However, AFP has limitations in sensitivity and specificity. Approximately 30-40% of HCC patients show no significant AFP increase, and some advanced cases may have normal AFP levels [13]. Elevated AFP can also occur in non-liver cancer conditions. To reduce false positives, imaging and additional markers (e.g., PIVKA-II and AFP-L3) should be used. For AFP-negative or low-expression patients, combining des-γ-carboxy prothrombin (DCP), AFP-L3, and γ-glutamyl transpeptidase (γ-GGT) improves diagnostic sensitivity to over 85% [14]. As a traditional biomarker of aHCC, AFP still has irreplaceable clinical value in efficacy evaluation, prognosis stratification and recurrence monitoring, but its limitations need to be made up by combined detection and new markers.

#### DCP as a Circulating biomarker

DCP, a protein induced by vitamin K absence or antagonist-II (PIVKA-II), is major serum biomarkers for the detection of HCC, increasing in 95% aHCC patients with a diameter greater than 5 cm [15]. DCP is also a surrogate marker for predicting the treatment response and prognosis during sorafenib therapy, due to its association with hypoxic HCC cells induced by the antiangiogenic effect of sorafenib. Regarding the baseline DCP levels, several studies demonstrated that a high DCP level (>1000 mAU/mL) before sorafenib treatment is an independent predictor of poor prognosis in patients with aHCC [16]. Most HCC patients exhibit an elevated level of DCP within 4 to 8 weeks of initiating sorafenib treatment. However, an early elevation of DCP levels following sorafenib treatment solely indicates tissue hypoxia and cannot serve as a predictor for treatment response or survival time due to the possibility of DCP elevation despite therapeutic efficacy. In summary, as a therapeutic and prognostic biomarker for aHCC, DCP exhibits high sensitivity and specificity. Notably, it is particularly effective in patients who are AFP negative or exhibit low AFP expression, enabling the effective prediction of treatment responses and the accurate assessment of prognostic risks. When combined with AFP detection, it can further enhance the accuracy of diagnosis and monitoring, thereby providing a critical foundation for the precise treatment of aHCC [17, 18].

#### Circulating angiogenesis-related biomarkers

Circulating angiogenesis-related biomarkers are critical exploration fields for systemic treatment of HCC, especially in tyrosine kinase inhibitor (TKI) treatment. These angiogenesis-related biomarkers are markedly associated with signaling cascades of tumor proliferation and neoangiogenesis, several of which are relevant targets of sorafenib and lenvatinib [19]. In the SHARP trial, the angiogenesis biomarkers angiopoietin-2 (Ang2) and vascular endothelial growth factor (VEGF) were independent predictors of survival but not predictors of treatment response to sorafenib in patients with aHCC [20]. However, most biomarker studies have focused on the therapeutic mechanisms of sorafenib due to its dominance in systemic treatment of aHCC. At present, limited studies have explored biomarkers for predicting the efficacy of lenvatinib and second-line targeted agents such as regorafenib. In the phase III REFLECT trial, high baseline levels of VEGF, fibroblast growth factor 21(FGF21), and Ang-2 were prognostic factors for shorter OS in both lenvatinib and sorafenib groups [20]. In the phase III RESORCE trial, decreased baseline concentrations of Ang-1 and four other evaluated proteins were significantly associated with increased OS after regorafenib treatment [7]. It have moderate sensitivity and low specificity in aHCC, and are mainly used to evaluate the efficacy of anti-angiogenic therapy and predict the prognosis of patients [20, 21]. Although its diagnostic efficacy alone is limited, its combination with imaging and other biomarkers, such as AFP and DCP, can improve the accuracy of the assessment of tumor angiogenic activity and response to therapy.

#### Cytokines and chemokines as Circulating biomarkers

Inflammatory cytokines and chemokines coordinate the tumor microenvironment, and the inflammatory milieu may either promote or impede cancer progression [22]. IL-6 is implicated in various hepatic pathologies, particularly in liver regeneration and oncogenesis. High IL-6 levels as a novel prognostic biomarker were significantly correlated with shorter PFS and OS in aHCC patients receiving atezolizumab plus bevacizumab [23]. IL-17 A is a well-established proinflammatory cytokine. The results of a retrospective suggested that higher serum IL-17 A levels (> 1.94pg/m) were a promising prognostic biomarker for predicting poor PFS in patients with HBV-related aHCC treated with sorafenib [24]. The infiltration of T cells in the tumor immune microenvironment shaped by TGF-β is diminished, indicating a potential correlation between elevated plasma levels of TGF-β and reduced efficacy of immunotherapy. In a phase II trial of pembrolizumab, the levels of plasma TGF-β were found to be lower in responders compared to non-responders and plasma TGF-β ≥ 200 pg/ml was significantly associated with shorter PFS and OS [25]. Nevertheless, cytokines and chemokines (such as IL-6, IL-8, CXCL10), as therapeutic and prognostic biomarkers of aHCC, have moderate sensitivity and low specificity. These biomarkers are primarily utilized to assess the inflammatory status of the tumor microenvironment and the efficacy of immunotherapy responses [17, 26].

#### Systemic inflammatory ratios as Circulating biomarkers

Systemic inflammatory ratios, such as neutrophil to lymphocyte ratio (NLR) and platelet to lymphocytes ratio (PLR), predict the response and prognosis of aHCC patients by reflecting inflammation and immune status [27]. The mechanism is related to inflammation-driven tumor progression and immunosuppression. A large exploratory analysis of two phase III trials enrolling 827 HCC patients reveled that high NLR (> 3.1) was a strong prognostic factor for poorer OS in both sorafenib and placebo cohorts [28]. In terms of ICI therapy, decreased NLR in patients with HCC treated with anti PD-1 therapy was correlated with a better treatment response and improved OS [29]. Two trials also found lower NLR was identified as a contributing factor to the response of nivolumab and pembrolizumab [10]. Moreover, a low NLR was correlated with longer OS in patients with unresectable HCC treated with atezolizumab plus bevacizumab [30]. Similarly, a high PLR served as an inflammatory marker highly correlated with poor prognosis in patients with HCC [29]. Although the diagnostic efficacy is limited when used alone, the combination of NLR and PLR with clinicopathological features can improve the predictive accuracy of response and prognosis.

#### Circulating immune cells

Various circulating immune cells in peripheral blood associating with the response and prognosis have been extensively evaluated as predictive biomarkers, especially the peripheral blood mononuclear cells (PBMCs) reflecting the landscape and dynamic change of tumor-associated immune cells [31]. Pretreatment PBMCs are a valuable predictor of systemic therapies efficacy. Kalathil SG et al. first proved immunomodulatory effects of sorafenib treatment on PD-1 + T cells and T regulatory cells [32]. Another study demonstrated that the frequency of CD4 + PD-1 + cells in PBMC was higher in among responders to tremelimumab [33]. Samuel Chuah et al. found that the higher frequencies of peripheral Tregs, CD11c + antigen-presenting cells and CXCR3 + CD8 + effector memory T cells were significantly associated with superior PFS in patients with HCC receiving immunotherapy [34]. In terms of combination therapy, the outcoms of the exploratory study demonstrated that the lower frequency of pretreatment peripheral naïve CD8 + T cells could be more likely to respond to treatment in patients with the cohort of lenvatinib plus an anti-PD-1 antibody [35]. In conclusion, circulating immune cells serve as a valuable biomarker for predicting the response and prognosis of patients with HCC to immunotherapy. By reflecting the dynamic changes in systemic immune status and tumor immune microenvironment, these cells are closely associated with anti-tumor activity and immune escape mechanisms. Despite individual variations and differences in detection methods, it exhibits moderate performance of sensitivity and specificity.

### Liquid biopsy

Liquid biopsy has gained great attention in cancer management over the past decade due to its ease of acquisition, minimally invasive procedure and sequential availability compared to traditional tissue biopsy [36, 37]. It should be noted that a solitary biopsy specimen with limited tumor tissue may not accurately reflect the entire tumor due to the marked heterogeneity of HCC. Therefore, exploring biomarkers from liquid biopsy seems more rational. The most widely explored analytes are circulating tumor cells (CTCs), circulating tumor DNA (ctDNA), micro RNAs(miRNAs).

#### CTCs as a liquid biopsy

CTCs are the “seed” of a tumor and contain a comprehensive set of information involved in DNA, RNA, proteins, and metabolites. The number of CTCs has been found to exhibit a positive correlation with disease stage, metastasis, and AFP levels in HCC. The PD-L1 expression level on CTCs may serve as a clinically applicable biomarker for immunotherapy, and its dynamic changes could potentially predict the therapeutic response. Yue et al. confirmed that the abundance of PD-L1high CTCs at baseline was significantly associated with favorable treatment response and PFS in HCC patients receiving anti-PD-1 therapy [38]. Recent studies also confirmed that PD-L1 expression in CTCs may have utility in predicting favorable immunotherapy response and prognosis in HCC patients [39]. CTCs exhibit the advantages of being non-invasive, enabling dynamic monitoring, and offering high resolution, thereby providing a critical foundation for personalized treatment and prognosis evaluation. However, this approach exhibits limitations such as inadequate standardization of detection technology, elevated costs, and reduced sensitivity in patients with low CTC burdens. Further standardization and validation are necessary to facilitate its clinical application.

#### CtDNA as a liquid biopsy

ctDNA can reflect the gene mutation, tumor burden and treatment response of aHCC in real time by detecting the DNA fragments in peripheral blood. Its mechanism is closely related to the genomic instability and clonal evolution of tumors. In HCC, the levels of ctDNA are positively associated with tumor size, vascular invasion, distant metastasis and tumor recurrence [40]. ctDNA demonstrates its potential in predicting pathologic response and recurrence after combination treatment [40]. A phase II study recruited 18 patients with aHCC to receive carrelizumab combined with apatinib as neoadjuvant therapy. More mutations at baseline were observed in patients with pathological complete response or major pathological response. Patients with ctDNA positive after neoadjuvant therapy exhibited a tendency towards shorter recurrence-free survival compared to those with ctDNA negative. An exploratory study longitudinally detected ctDNA for monitoring efficacy of atezolizumab plus bevacizumab in 45 patients with unresectable HCC in GO30140 arm A. Patients with higher baseline levels of ctDNA were found to have a greater initial tumor burden, while patients with ctDNA negative after 3 cycles of combination treatment showed longer PFS compared with those with ctDNA positive [41]. The most significant attributes of ctDNA include its high sensitivity, non-invasive nature, and precise analysis of tumor genomic variations, providing a critical foundation for personalized treatment strategies, efficacy monitoring, and prognosis evaluation in aHCC. However, the high heterogeneity of ctDNA in the circulatory system also limits their utility as predictive biomarkers. Further, the integration of novel multi-omics tools may yield valuable insights into critical clinical inquiries.

#### Circulating MiRNAs as a liquid biopsy

miRNAs, a type of non-coding and single-stranded RNA molecules with approximately 22 nucleotides in length, play pivotal roles in regulating cell proliferation and development, as well as the pathogenesis of liver cancer [42]. It exhibits high stability, are non-invasive, and accurately reflect the biological behavior of tumors [42]. Notably, an exploratory study analyzed plasma and tumor samples from RESORCE trial to identify circulating biomarkers and genetic features of tumors associated with response to regorafenib [7]. The results demonstrated that the level of 9 plasma miRNAs (miR30A, miR122, miR125B, miR200A, miR374B, miR15B, miR107, miR320, and miR645) are significantly associated with OS after regorafenib treatment [7]. These studies have investigated multiple potential miRNAs for predicting response to TKI treatment, yet their role in immunotherapy and combination therapy remains unexplored.

### Tumor tissue-driven biomarkers

#### Programmed cell death ligand 1 (PD-L1) expression

PD-L1 expressed on both antigen-presenting cells and tumor cells interacts with immune-checkpoint proteins to negatively regulate the adaptive antitumor immune response [43]. It can directly reflect the status of the tumor immune microenvironment and is closely associated with the efficacy of immunotherapy. However, PD-L1 expression exhibits significant heterogeneity, and there remains a lack of complete standardization in detection methods and threshold criteria [43]. Firstly, there is currently no consensus on which cell subsets to evaluate for PD-L1 expression in the KYENOTE 224 trial [44]. Secondly, the predictive value of PD-L1 expression remains unclear. In the phase III CheckMate 459 trial, PD-L1 positive tumors showed a better response to nivolumab compared to sorafenib, despite the nivolumab didn’t improve the OS as a first-line treatment for HCC [45]. In contrast, the CheckMate 040 trial demonstrated that the ORR were independent of PD-L1 expression on tumor cells, although higher levels of tumor PD-L1 expression were associated with improved OS in HCC patients receiving nivolumab [10]. Therefore, the absence of PD-L1 expression should not serve as a criterion to preclude aHCC patients from receiving anti-PD-L1 antibodies. Based on the IMbrave150 trial data, atezolizumab plus bevacizumab was approved by the FDA for patients with unresectable or metastatic HCC who had not received prior systemic therapy, regardless of their PD-L1 status [46]. The earlier phase Ib GO30140 trial as well as other studies also did not did not incorporate PD-L1 status as an enrollment criterion for patient. In summary, the predictive value of PD-L1 status for efficacy of anti-PD-L1 antibodies or combination therapies should be prospectively investigated in the future.

#### Tumor microenvironment (TME)

The efficacy of systemic therapies relies on the composition of TME. T-cell infiltration within the TME is a prerequisite for immune checkpoint blocking, especially CD8 + T cells, have been shown to play a critical role in controlling tumor progression. The CheckMate040 trial found that higher densities of CD3 + or CD8 + TILs exhibited a trend towards improved OS, but CD4 + or FOXP3 + TILs were not associated with a survival benefit [10]. Moreover, the outcomes demonstrated that responders had a higher density of infiltrating CD8 + T cells, CD3 + T cells and GZMB + CD3 + T cells in tumor areas than nonresponders in GO30140 arm A cohort. In the same way, patients with a high density of intratumoral CD8 + T cells showed longer OS and PFS with atezolizumab plus bevacizumab compared with sorafenib in the IMbrave150 cohort [13]. Macrophages with two main subtypes are also crucial components of the TME. In a phase Ib clinical trial, a total of 50 aHCC patients received sintilimab plus IBI305. The researchers found that patients with a high infiltration of M1 macrophages (CD68 + CD163-) not only showed a significantly prolonged PFS and OS as previously reported, but also demonstrated a markedly improved treatment response to combination therapy [47]. Spatial transcriptomics (e.g., TIMES system) combined with multi-marker analysis provide high-resolution information on the heterogeneity of TME. By integrating the information of immune cell composition, molecular expression, metabolic characteristics and spatial distribution, it provides a multi-dimensional basis for individualized treatment and accurate prognosis evaluation.

#### Tumor mutational burden (TMB)

TMB, a metric of cancer mutation count, positively correlates with the abundance of neo-antigens and thus increases the likelihood of self neo-antigen immunogenicity and T cell activation [47]. In HCC, the correlation between TMB and the efficacy of immunotherapy has not been fully clarified. Some studies have indicated that patients with high TMB benefit more from immunotherapy, while some studies have reported no significant correlation between TMB and treatment outcomes. A meta-analysis including 29 studies with 5396 HCC patients explored the benefits of combination therapy with ICIs and predictive role of TMB in HCC.In individualized analysis, patients with high-TMB (> 10 mutations/Mb) exhibited superior 1-year OS rates (28% versus 15%, *P* = 0.025) compared to those with moderate-TMB (1–10 mutations/Mb) [48]. TMB is generally observed to be low in HCC, which might potentially restrict its predictive value for treatment response in HCC patients [49]. In the GO30140 and IMbrave150 trials, the median TMB assessed by two detection methods was 5.6 mutations/Mb and 4.4 mutations/Mb, respectively.In GO30140 arm A group, patients with high TMB only showed a longer PFS than low or moderate TMB, although the difference was not statistically significant. And there was no discernible correlation between TMB and response rate or survival benefits in the atezolizumab plus bevacizumab cohort of IMbrave 150 [13]. In the future, it will be essential to investigate the integration of TMB with other biomarkers (e.g., PD-L1 and gene mutation profiles) to enhance predictive accuracy, particularly in optimizing treatment strategies for patients with high TMB.

#### Specific gene mutations

The most prevalent somatic mutation in HCC is the telomerase reverse transcriptase (TERT) promotor mutation (60%), which is associated with up-regulation of telomerase activity and promotes unlimited proliferation of tumor cells [2]. The second most frequently mutated gene is CTNNB1 (30%) with characteristic of immune-excluded which activates the WNT/b-catenin signaling pathway [2]. Other important mutations include TP53 (25%), AXIN1 (10%) and genes encoding epigenetic regulators [4]. However, the most prevalent mutations involved in TERT, CTNNB1 and TP53 mutations are currently undruggable. Therefore, recapitulating the genomic and phenotypical heterogeneity of HCC patients has been crucial for target identification [4]. The mutation landscape derived from patients with HCC enrolled in IMbrave150 confirmed frequent disease-associated somatic mutations in HCC [13]. Evaluation of several frequently mutated genes revealed a potential correlation between the mutation status of CTNNB1 and TERT promoters and clinical outcomes. The survival benefit of atezolizumab plus bevacizumab was more obvious in patients with TERT mutation than in those with TERT wild-type tumors [13]. For 31 patients treated with ICIs, activation of WNT/β-catenin signaling pathway was associated with poor treatment response and survival benefit compared to those with wild-type HCC [50]. CTNNB1 mutation is associated with a non-T-cell inflammatory tumor microenvironment and may reduce sensitivity to ICI monotherapy. Ogawa K et al. explored the effect of atezolizumab plus bevacizumab in patients with HCC harboring CTNNB1 mutation. They discovered that among 33 HCC patients treated with atezolizumab in combination with bevacizumab, 24.2% exhibited CTNNB1 mutations [51]. No significant differences were observed between wild-type and mutant CTNNB1 patients in terms of ORR, PFS, and OS. These findings indicate that the inclusion of bevacizumab with atezolizumab may potentially counteract the immunosuppressive effects associated with CTNNB1 mutations, thereby preserving comparable treatment efficacy between the groups. TERT and CTNNB1 mutations served as potential biomarkers have prognostic and predictive value in aHCC, but play a limited role in combination therapy.

#### Specific gene signatures

Transcriptomic profiling data from HCC tumor tissues has enabled the delineation of the TiME and identification of signatures that are correlated with clinical response and survival benefits to systemic therapy. Currently, a novel prognostic model utilizing five immune-related signatures (LDHA, PPAT, BFSP1, NR0B1, and PFKFB4) has been developed to predict the efficacy of immunotherapy in patients with HCC, enabling stratification of candidates who are likely to respond positively to such treatment [52]. The CheckMate 040 trial revealed that an inflammatory signature consisting of CD274, CD8α, LAG3, and STAT1 was associated with both improved ORR and OS in both the dose-escalation and dose-expansion phases, suggesting that underlying inflammation within the TME may contribute to favorable clinical outcome [10]. In the pivotal GO30140 and IMbrave150 trials, pre-existing immunity including T-effector signature was associated with better clinical outcomes with the atezolizumab plus bevacizumab group [13]. The high expression ratio of Tregs to effector T cells was associated with a reduced clinical benefit. Furthermore, the combination therapy demonstrated enhanced survival benefits compared to atezolizumab monotherapy, particularly in patients with high expression of KDR, Tregs and myeloid inflammation signatures [13]. Specific gene expression is a potential tool for molecular typing and individualized treatment of HCC, but its detection cost is high and requires high-quality tumor tissue or blood samples. Conducting extensive bioinformatics analysis on renowned large databases to identify predictive gene signatures, and subsequently validating these findings through preclinical studies and clinical trials, may prove to be an effective solution.

### Gut microbiota

Gut microbiota influences the response to immunotherapy in HCC through metabolites (such as short-chain fatty acids) and immune regulatory mechanisms [53]. The dynamic variations in the gut microbiome could potentially serve as early predictors of immunotherapeutic outcomes in HCC. A prospective study enrolled eight HCC patients treated with nivolumab as a second-line systemic treatment to investigate the correlation between gut microbiome composition and prognosis of immunotherapy. A higher abundance of Clostridia, Prevotella and Ruminococcaceae was observed in the responder group, whereas Ruminococcus gnavus was predominant in the non-responder group [54]. A recent study concluded that a favorable gut microbiota composition and low intestinal inflammation are associated with disease control in HCC patients treated with tremelimumab and/or durvalumab. The levels of pretreatment fecal calprotectin and serum PD-L1 were found to be significantly lower in responders compared to nonresponders [55]. Moreover, dysbiosis of the gut microbiota, including reduced diversity, has been associated with an unfavorable prognosis in patients with aHCC. Modulation of the intestinal flora, such as probiotics and fecal microbiota transplantation, may enhance the anti-tumor immune response and thereby improve patient outcomes [56]. It should be noted that the gut microbiota can be influenced by multiple factors of environment, diet, and lifestyle, all of which have the potential to impact the immune system and subsequently regulate the response to ICIs [56].

### Artificial Intelligence-enabled imaging biomarkers

Artificial intelligence-based radiomics and deep learning represent an emerging field that transforms standard-of-care medical imaging into mineable, high-throughput, and quantitative features through the application of multiple image-characterization algorithms [57]. As a digital biopsy, it also offers a non-invasive, repeatable and comprehensive insight into tumor biology and heterogeneity without additional blood and tissue samples [57]. A retrospective study proposed a radiomic model based on contrast-enhanced CT with 58 aHCC patients from a single clinical center to predict the response to anti-PD-1 antibody monotherapy. The radiomic model, comprising eight radiomics features extracted from intra- and peri-tumoral regions, shows excellent predictive performance in both training cohort (AUC 0.894) and validation cohort (AUC 0.883), respectively [58]. Notably, another study is the first to demonstrate that tumor radiomic features based on pretreatment MRI can serve as predictors of objective response to lenvatinib plus an anti-PD-1 antibody therapy in unresectable or aHCC patients, providing additional predictive value beyond clinicopathologic features [59]. The model also underwent validation in multiple clinical centers, revealing that radiomic features were significantly associated with PFS and OS following the initiation of this combination therapy. Imaging biomarkers offer the advantages of non-invasiveness, high resolution, repeatability, and precise analysis of tumor heterogeneity. However, model training is dependent on high-quality labeled data, and the generalization capability across different devices and centers requires further validation.

### Multimodal biomarker strategies

The discovery and validation of the aforementioned biomarkers have substantially enhanced both the understanding and management of aHCC.However, the translation of individual biomarkers into clinical practice remains challenging due to the heterogeneity of HCC and the complexity of its molecular landscape. To address these challenges, multimodal biomarker strategies, which integrate multiple types of biomarkers, have emerged as a promising approach to improve diagnostic accuracy, prognostic stratification, and treatment selection.

In aHCC, individual biomarkers such as AFP have long been used for diagnosis and monitoring. However, AFP alone lacks sufficient sensitivity and specificity, particularly in non-viral HCC cases. To overcome these limitations, composite biomarkers that combine AFP with other molecular signatures, such as genomic, proteomic, or metabolomic profiles, have been explored [60, 61]. For example, integrating AFP with ctDNA mutations or epigenetic alterations can enhance the detection of early recurrence and provide insights into tumor evolution under therapeutic pressure [62]. Similarly, combining AFP with inflammatory markers like C-reactive protein or IL-6 may improve prognostic stratification by reflecting both tumor burden and systemic inflammation, which are critical drivers of HCC progression [23, 63, 64]. Another promising multimodal strategy involves the integration of imaging biomarkers with molecular data. Radiomic features derived from contrast-enhanced CT or MRI, such as tumor texture, vascularity, and heterogeneity, can be combined with serum or tissue-based biomarkers to create comprehensive predictive models. For instance, radiomic signatures reflecting tumor hypoxia or immune infiltration, when paired with immune-related gene expression profiles, may predict response to ICIs in aHCC [65]. This approach not only enhances the predictive power of biomarkers but also provides a non-invasive means to monitor treatment response and resistance. Furthermore, the incorporation of liquid biopsy-based biomarkers, such as exosomal miRNAs or CTCs, into multimodal strategies offers a dynamic and real-time assessment of tumor biology [66]. Similarly, CTC enumeration and characterization, integrated with genomic and proteomic data, can provide insights into metastatic potential and guide personalized treatment strategies [66].

Multimodal biomarker strategies represent a paradigm shift in aHCC management. These approaches integrate diverse biomarkers to overcome single-marker limitations and enhance understanding of tumor biology. Future research should validate these strategies in large prospective cohorts and explore their utility in precision medicine to improve outcomes for patients with aHCC.

## Evolving biomarker-driven systemic therapies

### Biomarker-driven immunotherapies

#### Adoptive cell therapy (ACT)

**Fig. 4:**
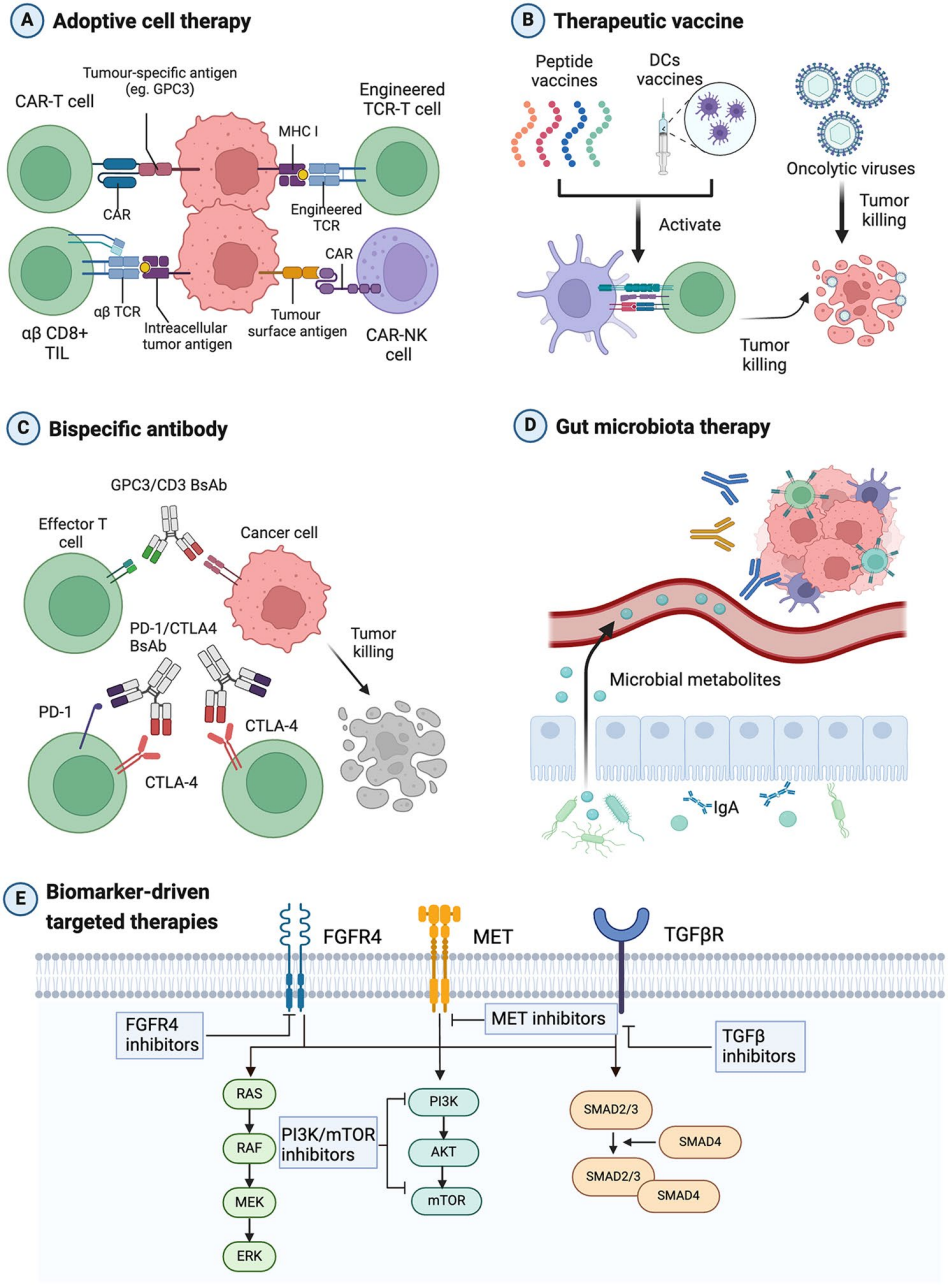
Emerging biomarker-driven therapies with therapeutic potential in HCC. Currently, emerging therapies driven by biomarkers in HCC include adoptive cell therapy (**A**), therapeutic vaccines (**B**), bispecific antibodies (**C**), gut microbiota therapy (**D**) and novel target therapies(**E**). ACT, adoptive cell therapy; CAR T cell, chimeric antigen receptor T cell; NK cell, natural killer cell; GPC3, glypican 3; TCR, T cell receptor; MHC, major histocompatibility complex; DC, dendritic cell; BsAb, bispecific antibody; CTLA4, cytotoxic T lymphocyte-associated protein 4; FGFR, fibroblast growth factor receptor; TGFβR, transforming growth factor-β receptor

ACT, enhancing the anti-tumour immune response via infusion of immune cells, is the most representative non-ICI immunotherapy in HCC [2] (Fig. 4). Chimeric antigen receptor T cell can recognize the antigens on the tumor surface to eliminate tumor cells and avoid immune escape [16] (Fig. 4A). Based on specific biomarkers and targets, CAR -T therapy for HCC is in development. Recently, several clinical trials successfully assessed the safety and efficacy of CAR-T cells targeting GPC3, with some encouraging preliminary results [67]. Moreover, multiple CAR T cell therapies targeting c-MET, NKG2DL, CD147, CD133 and MUC1 are being tested for the treatment of HCC in clinical practice [16]. TCR-engineered T cells have a high affinity for both surface and intracellular antigens by specifically recognizing the tumor antigen peptides-MHC complex with greater applicability in solid tumors including HCC [4]. Currently, TCR-T therapies targeting HBV or HCV antigens and AFP has been initiated in clinical trials, however, this treatment will inevitably lead to off-target toxicity due to high binding promiscuity of TCR [2, 3].

#### Therapeutic vaccines

The application of therapeutic vaccines is a promising biomarker-driven strategy to enhance specific immune responses by introducing tumor antigens. It mainly include peptide, dendritic cell and oncolytic viruses (Fig. 4B). Based on well-defined tumour-associated antigens, peptide vaccines driven by AFP, GPC3 was well-tolerated and elicited high rate of spontaneous T cell responses [68]. Dendritic cells (DCs) are one population of the specialized APCs and are responsible for T-cell stimulation and immune response inducement. The safety and feasibility of DC vaccine have been validated [69]. Oncolytic viruses are viral particles engineered to lyse tumor cells and induce anti-tumor immune responses. JX-594 (Pexa-Vec), a main oncolytic virus, showed survival benefit and manageable safety in phase II trial [70]. Currently, two phase III clinical trials of JX-594 for the treatment of aHCC are being further explored.

#### Bispecific antibody

Bispecific antibodies exhibit dual specificity by binding to two different epitopes on the same or different antigens, enabling them to exert activities beyond those of natural antibodies (Fig. 4C). This unique feature offers numerous opportunities for therapeutic applications targeting soluble or cell-surface antigens. Currently, BsAbs developed for HCC including PD1/CTLA4 (e.g. AK104), PD-L1/CTLA4 (e.g. KN046), PD-L1/LAG3(e.g. Tebotelimab) and GPC3/CD3 [4]. Preliminary data suggested that the combination of AK104 and lenvatinib has shown promising anti-tumor efficacy and an acceptable safety profile as a first-line therapy for unresectable HCC. The ORR and DCR was 44.4% and 77.8%, respectively, among the cohort of 18 patients with uHCC who underwent evaluation for antitumor activity following treatment with AK104 and lenvatinib [71]. Other BsAbs have also demonstrated promising early results in both preclinical and clinical investigations for HCC [2–4]. Currently, more dual and multi-antibody technologies need to be continuously improved while disclosing relevant data.

#### Gut microbiota-related immunotherapy

It is increasingly evident that the modulation of gut microbiota can serve as a novel and significant adjunct to anti-cancer therapeutic modalities based on immune-related approaches [2, 3] (Fig. 4D). Given the profound dysbiosis in HCC patients with underlying cirrhosis, it’s tempting to speculate that gut microbiome modulation may have a more significant impact on HCC than other tumors and contribute to immunotherapy failure in some cases [34]. However, the role of the gut microbiome in antitumor immune responses from HCC patients remains unclear to date. A clinical trial (NCT03785210) combining vancomycin treatment with ICIs may hopefully answer whether combining the selective manipulation of the gut microbiota and immunotherapy will be beneficial for HCC patients [2].

### Biomarker-driven targeted therapies

#### TGF-β Inhibition

TGF-β has been shown to be a predictive biomarker for ICI therapy of HCC. Interestingly, the activation of TGF-β pathway also has dual and opposite effects during tumor development. In early tumor stage, it can inhibit proliferation, while it promotes cell invasion, angiogenesis, metastasis, and drug resistance at late stage. In addition, recent studies showed that TGF-β could be resistant to PD-1 blockade by exhausting immune signatures and excluding T cells [72]. Galunisertib, a TGF-β receptor 1 inhibitor as a second-line therapy for HCC has been investigated, and the study reported that patients with low AFP levels had longer OS than those with high AFP (16.8 versus 7.3 months) [73] (Fig. 4E). The combination of galunisertib with sorafenib was also further explored [73]. Moreover, TGF-β pathway inhibition as a biomarker-driven therapies may be further combined with PD-1/PD-L1 inhibitors, or using bifunctional fusion proteins blocking both TGF-β and PD-L1 [74].

#### MET inhibitors

MET, a tyrosine kinase receptor, has a single ligand, hepatocyte growth factor. Activation of the MET/HGF axis is associated with the development, progression and drug resistance of HCC [75]. MET inhibitors, including four non-selective inhibitors and two selective inhibitors have been explored for their efficacy in HCC [76] (Fig. 4E). Non-selective MET inhibitors exert their antitumor activity by blocking non-MET targets. However, selective inhibitors for MET-high HCC, are more likely to produce effective MET inhibition and reduce off-target toxicity [76]. And the selective MET inhibitors such as tepotinib and capmatinib have exhibited superior efficacy and manageable safety in patients with MET-high HCC in several clinical trials [77].

#### FGFR4 inhibitors

Blocking the FGF19/FGFR4 axis is another promising biomarker-enabled therapy in HCC because the activation of this pathway is closely related to HCC cell invasion (Fig. 4E). FGF19 also played an important role in sorafenib resistance [78]. In a phase I trial, fisogatinib (BLU-554), a selective FGFR4 inhibitor, resulted in a 17% ORR in the FGF19 + group and 0% ORR in the FGF19- group. The trial indicated that targeting the FGF19/FGFR4 pathway can be used as a potential biomarker-driven approach, and it also highlighted that FGF19 could serve as a biomarker for patient selection [78]. Currently, the efficacy of fisogatinib is being further explored as a single agent in HCC.

#### mTOR inhibitors

The PI3K–AKT-mTOR pathway is an important signaling axis for the development and progression of HCC [79] (Fig. 4E). Thus, inhibiting these pathway may be served as a novel therapeutic strategies. However, several mTOR inhibitors, such as everolimus and temsirolimus, have been proposed as monotherapies for HCC but have failed in clinical trials [79]. Some new mTOR inhibitors that have broader inhibitory effects on the PI3K–AKT–mTOR pathway, such as onatasertib, are being explored in early-stage clinical trials for aHCC. Current biomarker-driven strategies are also exploring the combination therapies that inhibit compensatory signalling pathways that are suspected to cause therapy resistance.

## Conclusion

With the approval of multiple first- and second-line agents, especially the combination therapies based on ICI regimens, the landscape of systemic therapy for aHCC is more diverse than ever before. The efficacy of current systemic therapies shows great heterogeneity in patients with aHCC, thereby identifying biomarkers for response prediction and patient stratification has become an urgent need. The complexity of defining biomarkers for systemic treatment strategies in HCC has been insufficiently studied, and no biomarker is currently suitable for clinical decision-making. The inherent heterogeneity of this disease in terms of clinical, molecular, and etiologic factors, coupled with the rapid evolution of its treatment landscape, presents significant clinical challenges. In terms of biomarker sources, blood-derived biomarkers are a desirable and non-invasive alternative for advanced patients when tissue biopsies may not be feasible from aHCC patients. More importantly, performing tissue biopsy prior to initiating systemic therapy already was recommend by the current clinical guidelines for the management of aHCC. This further facilitates a paradigm shift in the HCC management and potentially augments the future availability of tumor tissue. The exploration of future systemic therapeutic biomarkers is moving towards diversification of molecular markers, dynamic detection methods, and multi-omics prediction models.

## Data Availability

No datasets were generated or analysed during the current study.

## Abbreviations

HCC: Hepatocellular carcinoma
aHCC: Advanced hepatocellular carcinoma
ICIs: Immune checkpoint inhibitors
AFP: Alpha-fetoprotein
OS: Overall survival
PFS: Progression-free survival
DCP: Des-γ-carboxy prothrombin
TKI: Tyrosine kinase inhibitor
Ang: Angiopoietin
VEGF: Vascular endothelial growth factor
FGF: Fibroblast growth factor
TGF-β: Transforming growth factor-β
NLR: Neutrophil to lymphocyte ratio
PLR: Platelet to lymphocytes ratio
PBMCs: Peripheral blood mononuclear cells
CTC: Circulating tumor cells
ctDNA: Circulating tumor DNA
PD-L1: Programmed cell death ligand 1
TME: Tumor microenvironment
TMB: Tumor mutational burden
ACT: Adoptive cell therapy
